# Preparation of Nano/Micro Bimodal Aluminum Powder by Electrical Explosion of Wires

**DOI:** 10.3390/ma14216602

**Published:** 2021-11-02

**Authors:** Alexander Pervikov, Nikita Toropkov, Sergey Kazantsev, Olga V. Bakina, Elena Glazkova, Marat Lerner

**Affiliations:** 1 Institute of Strength Physics and Material Science, Siberian Branch of Russian Academy of Science, Av. Akademicheskii, 2/4, 634055 Tomsk, Russia; pervikov@list.ru (A.P.); zerogooff@gmail.com (N.T.); kzso@ispms.tsc.ru (S.K.); eagl@ispms.tsc.ru (E.G.); lerner@ispms.tsc.ru (M.L.); 2 Research and Education Center Additive Technologies, National Research Tomsk State University, 36 Lenin Ave., 634050 Tomsk, Russia

**Keywords:** PIM process, electrical explosion of wires, bimodal nano/micro powders

## Abstract

Electrical explosion of aluminum wires has been shown to be a versatile method for the preparation of bimodal nano/micro powders. The energy input into the wire has been found to determine the relative content of fine and coarse particles in bimodal aluminum powders. The use of aluminum bimodal powders has been shown to be promising for the development of high flowability feedstocks for metal injection molding and material extrusion additive manufacturing.

## 1. Introduction

Considerable progress in powder metallurgy can be obtained through a reduction in the powder particle size used down to nanosize. The development of materials using nanoparticles as building blocks is one of the most important future challenges for creating products with optimized or completely new properties [1].

Nanoparticles have unique mechanical and physical properties due to their large surface area-to-volume ratio and other peculiar traits [2]. The use of nanoparticles makes it possible to increase the strength, hardness, impact strength, and other important characteristics of bulk materials. Nanopowders exhibit more isotropic sintering characteristics, and the sintered parts have a lower surface roughness than that of the sintered parts made of micropowder [3]. Low sintering temperature due to low sintering activation energy is also one of the characteristic advantages of nanopowders [4,5].

However, nanoparticles tend to agglomerate, and nanoparticle agglomerates of nanoparticles behave like a separate large particle, which leads to the loss of their unique properties. Nanoparticle-based feedstocks for the powder injection molding (PIM) process, being a mixture of the powder and organic binder, for example, exhibit a low content of the dispersed phase and a significant increase in the viscosity of the feedstock [6,7]. Explosive oxidation of metal nanopowders and the high cost of nanomaterials are also limiting factors for their use in powder metallurgy processes.

However, the integration of nanoparticles with microparticles allows taking advantage of both nanoparticles and microparticles. Nano/micro particle composites, which most clearly manifest their advantages, are widely studied now. For example, the use of PIM feedstock based on stainless steel 316L bimodal powders containing 25 vol.% nanoparticles made it possible to obtain parts with a higher density than that based on micropowder [8].

The industrial application of powder mixtures containing two different particle size fractions (bimodal powders) has gained much attention in various fields of materials science, energy, and biotechnology. As a rule, bimodal powders are considered to be compositions containing two fractions—particles with a size of several micrometers and particles with a size of tens of micrometers in which the interparticle forces are small [9].

The use of bimodal powders in powder metallurgy leads to an improvement in the packing density [10,11]. This is due to the fact that small particles fill the voids between large particles. Such powders have less shrinkage during sintering and a higher density of the sintered material [12]. Additionally, the use of bimodal powders leads to an increase in apparent density. Thus, the addition of coarse powders to the 5 μm powder increases the apparent density by 12.7% (30 μm) and 5.6% (15 μm), improving the tap density by 5.6% (30 μm) and 4.9% (15 μm) [13]. Moreover, an increase in the density of the sintered material in binder jetting additive manufacturing [14] has been found when using multimodal, usually bimodal, powders [15,16,17,18,19].

Additionally, in the case of selective laser melting or direct metal laser sintering, the higher the density of the layer, the higher the density of the final part. The high density of the powder layer can provide faster production of parts without compromising their quality [20]. The use of powder alloy IN718 containing 83.75 vol.% fine fraction (D_10_–D_90_: 6.2–16.9 µm), and 16.25 vol.% coarse fraction (D_10_–D_90_: 26.5–50.5) when manufacturing parts by typical laser powder bed fusion resulted in a 10% increase in process productivity, higher tensile strength, and reduced mechanical anisotropy [21]. Additionally, the higher the layer density, the lower the surface roughness [22].

Nano/micro particle composites, as a rule, are obtained by mixing ready-made nano- and micropowders obtained separately in different processes [23,24,25]. However, when mixing powders, the particles of which differ significantly in density, size, and shape, the separation and disturbance of the homogeneity of the mixture are possible. In addition, the separate synthesis of nano- and microparticles requires bulky and expensive equipment that takes up large space and, as a rule, is difficult to operate and requires the involvement of a labor force with specialized knowledge [26].

Taking into account the above mentioned factors, promising technologies are those that ensure the in situ production of nano/micro particle mixtures with a controlled ratio of dispersed phases in one process. This approach enables reduction in the cost of nano- and microparticle composite manufacturing and ensures high homogeneity of the resulting mixtures, supplementing the existing manufacturing methods and creating completely novel nanocomposites. Electrical explosion of wires (EEW), the method that is usually used to obtain nanopowders of metals, metal oxides, and nitrides [27], has been proved to be versatile for the one-step preparation of nano/micro particle composites. Previously, the preparation of bimodal powders of SS 316L, Cr-60Ni-W, and Cr-70Ni-Al alloys for metal injection molding (MIM) technologies has been reported [28,29].

Aluminum is widely used in various powder metallurgy processes, including additive manufacturing [30,31], due to its high strength and low density. It is an ideal choice for applications requiring strong, lightweight components, such as in the aerospace and automotive industries [32].

This article presents the results of studies on the preparation of nano/microparticle composites by electrical explosion of aluminum wire. Dependences of the disperse composition of bimodal powders on the synthesis conditions were established. The flowability of model feedstocks based on bimodal electroexplosive aluminum powders was studied.

## 2. Experimental Procedure

**Figure 1:**
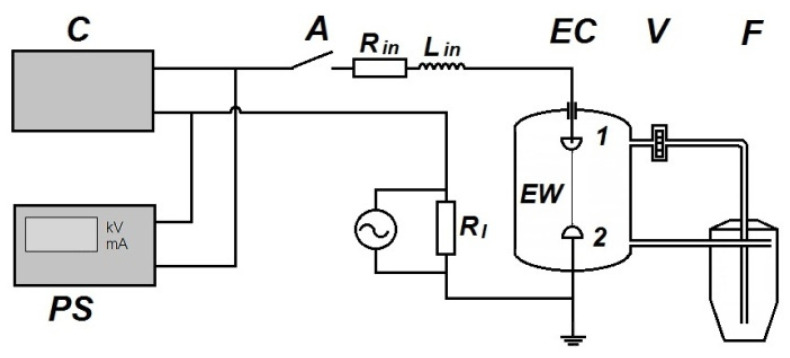
Schematic diagram of the EEW setup.

Bimodal aluminum powder was obtained by EEW of aluminum (0.35 mm in diameter, metal content 99.9%) wire on a setup the schematic diagram of which is shown in Figure 1. The wire coil was placed in a tight container, which was joined to the setup; the internal space of the setup was evacuated and filled with buffer gas argon under a pressure of 0.2 MPa. The experimental conditions are presented in Table 1.

The setup works as follows. High voltage source *PS* (Figure 1) charges the capacitive energy storage *C* to the given voltage *U*_0_, the value of which is monitored with a kilovoltmeter *kV*. Energy storage *C* consists of a bank of pulse capacitors connected in parallel. After switching on the arrester *A*, the energy stored in *C* is transferred to the wire *EW* located between the high-voltage electrode 1 and the grounded electrode 2 in the explosion chamber *EC*. The current *I(t)* flowing through the wire is recorded using the current sensor *R_I_*. The signal from the sensor is transmitted over a coaxial cable with a characteristic impedance of 75 ohms to a digital oscilloscope. *R_in_* and *L_in_* are intrinsic resistance and inductance of the setup electrical circuit, respectively. The energy level input into *EW* for the electrical explosion is controlled by the variation of high voltage *U*_0_.

When operating the setup, the gas is continuously circulated between the explosion chamber *EC* and the filter *F* by fan *V*. The aerosol (powder + Ar) enters the filter *F*, where the powder settles, and the gas purified from the powder goes back to *EC*.

Table 1 shows the wires’ dimensions (*d*—diameter, *l*—length), and operating parameters—*C, L*, and *U*_0_.

To determine the energy *E(t)* input into the wire, the expression (1) was used [33]:

(1) $$E\left(t\right)=U_{0\;}\int_{0}^{t}I\left(t\right)dt-\frac{1}{2C}\;{\left[\int_{0}^{t}I\left(t\right)dt\right]}^{2}-\frac{LI^{2}\left(t\right)}{2}-R\int_{0}^{t}I^{2}\left(t\right)dt$$

where *t* is the time of passage the current pulse *I(t)* through the wire, *U*_0_ is the voltage of the capacitive energy storage, *C* is the capacitance of the capacitive energy storage, and *L* and *R* are inductance and active resistance of the circuit, respectively.

The first term, on the right side of Equation (1), determines the energy transferred into the circuit; the second, third, and fourth terms of the equation determine capacitive, inductive, and active energy losses, respectively.

The circuit inductance *L* of the electric circuit was determined from the Equation (2) according results of the short circuit experiment, when the high-voltage and low-voltage electrodes were connected by a conductive bus of large section, with *T* being the period of the oscillatory discharge in the electric circuit.

(2) $$T=2\pi \sqrt{LC}$$

The relative value of the energy input into the wires (wire overheating) was determined as *E/E_s_* ratio, where *E* = *E(t)* is determined by the expression (1) and, *E_s_* is the aluminum sublimation enthalpy, being 33 J/mm^3^ [34].

The wire geometric dimensions (diameter and length) are known to affect the average particle size. When energies input *E/m* (*m*-wire mass) to wires and gas pressure in the explosion chamber are constant, the nanoparticle average size increases as the wire diameter *d* increases to a value *d* ≈ 0.3 mm [27]. In addition, the geometric dimensions of the wire directly determine the specific energy input into the wire. Therefore, in order to exclude the effect of wire geometric dimensions on dispersity of the particles obtained, wires of the same diameter and length were used in the experiments. Thus, the parameter affecting the dispersion composition of the powders obtained was only *E*.

**Figure 2:**
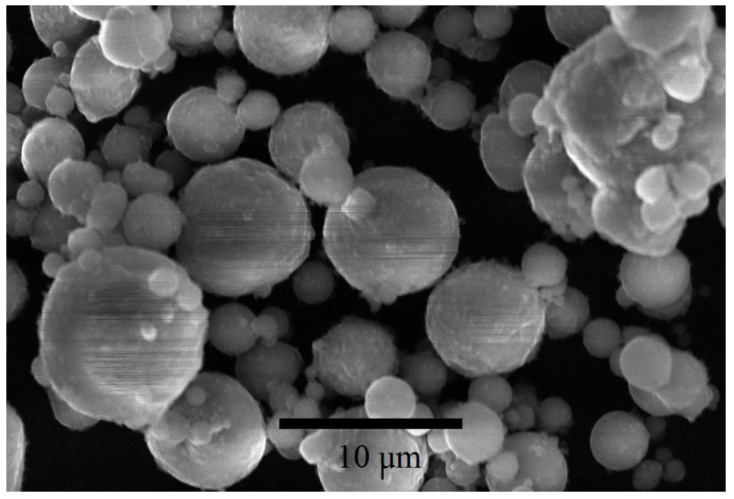
SEM-image of aluminum micropowder ASD-4.

Micron-sized aluminum powder grade ASD-4 (aluminum spherical dispersed powder) obtained by gas jet spraying of molten aluminum was supplied by the manufacturer (OOO SUAL-PM, Shelekhov, Russia). Particle size was less than 15 microns, with average particle size being about 7 microns (Figure 2).

The synthesized Al particles were characterized by transmission electron microscopy (TEM) using a JEM-2100 electron microscope (JEOL, Tokyo, Japan) and scanning electron microscopy (SEM) using a Quanta 200 3D electron microscope (FEI, Maribor, Slovenia).

To determine the average size of nanoparticles, according to electron microscopy data, histograms of the particle size distribution were constructed. To construct the histogram, sizes of at least 3000 particles were measured. The average particle size was determined by the expression *a_n_ = Σn_i_a_i_/Σn_i_*, where *n_i_* is the number of particles falling into the selected size range, and *a_i_* is the average particle diameter in the selected range.

Particle size distribution was determined using a CPS DC2400 disc centrifuge (CPS Instruments, Prairieville, LA, USA).

To prepare feedstocks, an MC2162 binder was used with 60 vol.% load of aluminum powders. The powdery binder was preliminarily mechanically mixed with aluminum powder, after which the mixture was passed through the ScientificLab LTEE26 extruder (Labtech Engineering Company Ltd, Samutprakarn, Thailand) 6 times at a temperature of 155 °C.

The determination of the feedstock melt flow index (MFI) was carried out by the plastometer IIRT-M (LOIP Ltd., St Petersburg, Russia). MFI was determined from the flow rate of molten material through a capillary nozzle at a standard load of 5 kg and a temperature of 155 °C in 3 parallel measurements.

## 3. Results and Discussion

**Figure 3:**
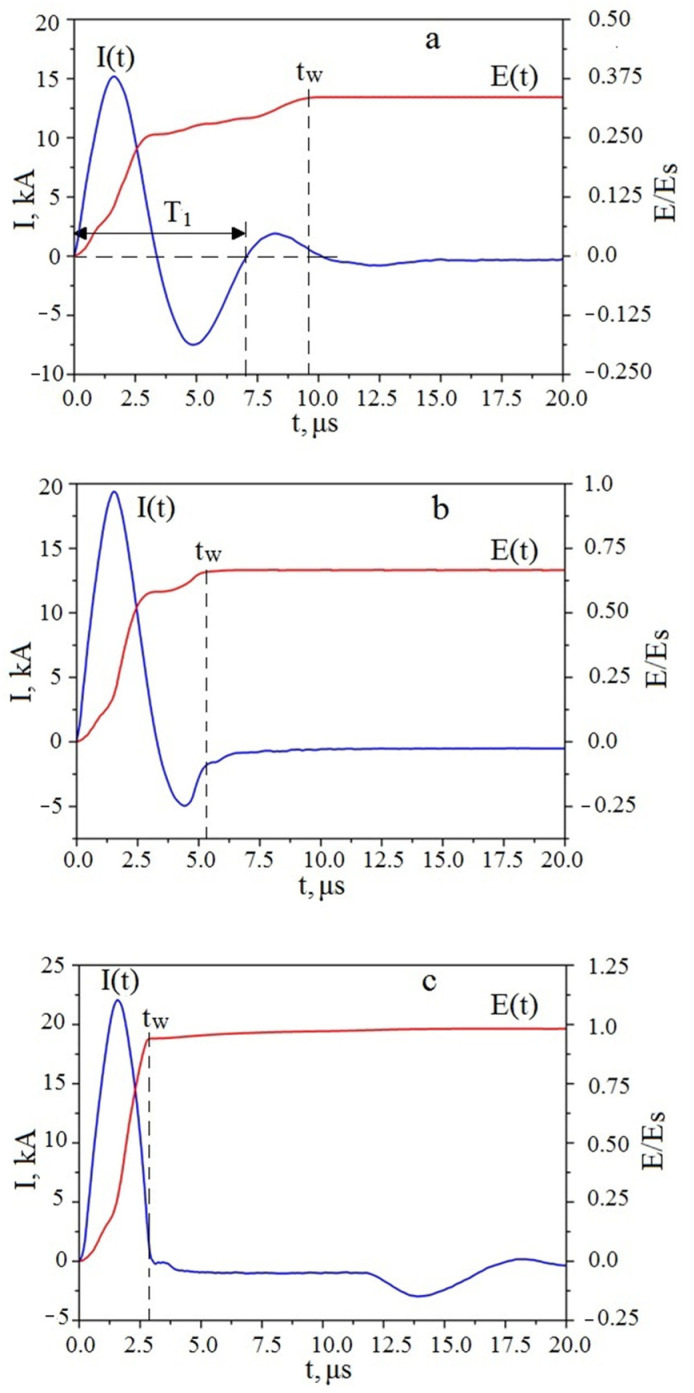
Temporal oscillogram curves of current *I(t)* and energy *E(t)* for electrical explosion of aluminum wires: (**a**) Sample 1, (**b**) Sample 2, (**c**) Sample 3.

Figure 3 shows *I(t)* oscillograms obtained at different energies *E(t)* input into the aluminum wires during electrical explosion of aluminum wires.

The point *t_w_* in Figure 3 denotes the time point when the transfer of energy to the wire stopped, that is, its heating ended. At this point, the metal of the wire was dispersed, which can be interpreted as an electrical explosion. It should be noted that the current *I(t)* oscillograms shown in Figure 3a,b is uncharacteristic for EEW. In accordance with expression (3), the circuit period *T* was 6.8 μs. The value of *T* was determined to be about 7.1 μs (Figure 3a). Thus, for Sample 1 the destruction of the wire took place in the third half-period of the discharge current *I(t)*, at the time point of 9.7 μs (Figure 3a). Additionally, for Sample 2, the destruction of the wire took place in the second half-period, at the time point of 5.3 μs. Sample 3 was obtained with the destruction of the wire in the first half period at a time point of 2.9 μs. Overheating value was in the range 0.33–0.95.

Based on values of current densities (Table 2), wire diameter (Table 1), and the data presented in Reference ([35], Figure 1), when synthesizing Samples 1 and 2, the conditions for uniform Joule heating of the metal were violated. The time of energy input into the wire exceeded the development time of microscopic magneto-hydrodynamic (MHD) sausage-type instabilities: *t_w_ > t_MHD_*.

The disperse composition of powders obtained using the EEW method depends on such parameters as the energy input into the wire, the duration of energy input into the wire, wire diameter and length, the composition (Ar, He, gas mixtures, or liquid), and the pressure of buffer gas. Dependences of the powder disperse composition on such parameters as wire diameter, wire length, and buffer gas pressure were reliably determined and described [27,34]. The choice of the wire geometric dimensions for different metals and their relationship with the circuit parameters are described in detail in [34]. It has been reliably established that production of nanopowders is achieved when energy input exceeds the sublimation energy of the metal in the mode of rapid wire explosion. At the same time, the dependence of the disperse composition of powders obtained in the *E/Es* < 1 mode has not been systematically investigated [36].

The phenomenon of the explosive destruction of metals in the form of wire heated by the current pulse occurs at *E/E_s_* > 0.2 ... 0.3 [37]. For the mode 0.2 < *E/E_s_* < 1, the condition *t_w_ > t_MHD_* (slow MHD mode [38]) is characteristic. The development of MHD instabilities is accompanied by the formation of local regions with smaller diameters (waists) along the wire length. In these regions, more intense energy release is observed as a result of Joule heating of the liquid metal by the current pulse, and the metal in these regions turns to the gas-plasma state [37]. Regions of hot liquid metal, enclosed between the waists, are dispersed in the form of liquid metal droplets, and the size of some droplets can exceed the wire diameter [39]. Since in the mode of slow EEW, the density of liquid metal under conditions of heating by current pulse does not exceed the density of liquid metal on binodal, the disperse composition of micron sized droplets formed during destruction of liquid metal regions will be determined by the value of specific energy input (J/(g∙μs)). The increase in specific energy input leads to an increase in the number of vapor seeds in a unit volume of liquid metal, which creates conditions to reduce the size of the liquid metal particles and the interval of particle size distribution [40]. The described phase transitions—namely, the transition of waist metal into the gas-plasma state and volumetric boiling of liquid metal—will determine the disperse composition of powders at conditions 0.2 < *E/E_s_* < 1.

**Figure 4:**
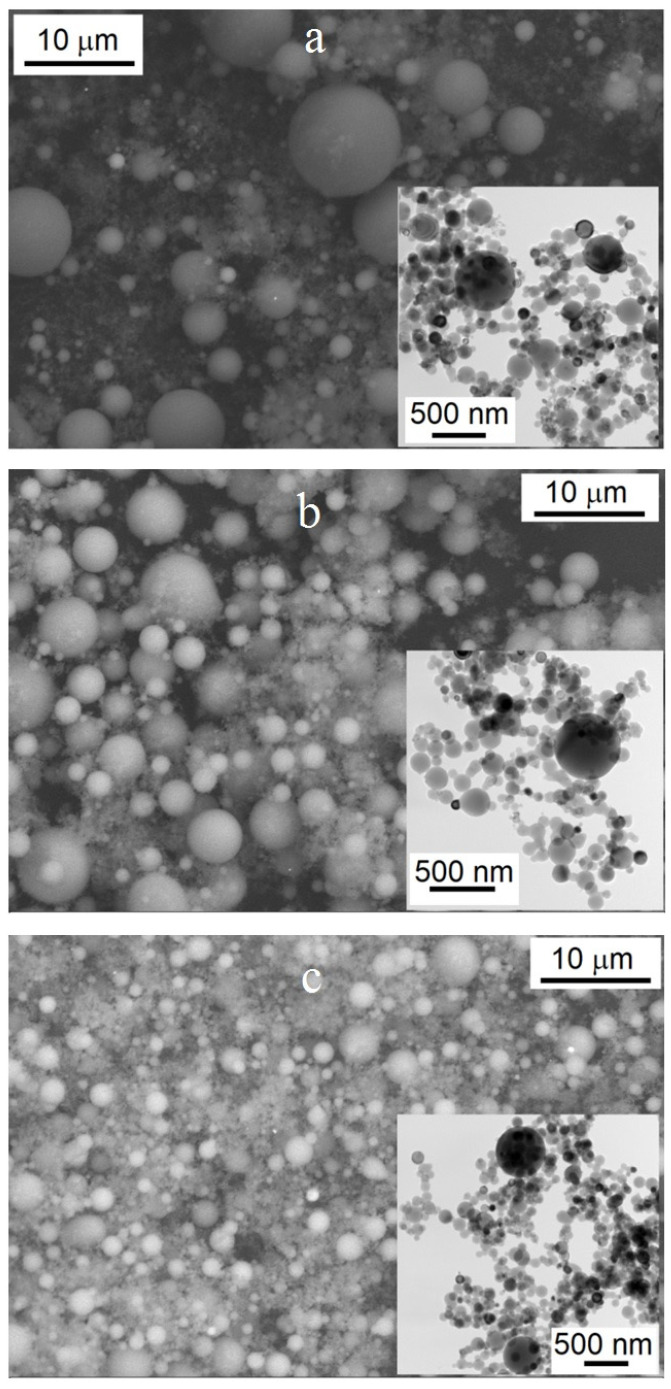
SEM images of aluminum particles. Inset shows TEM images of aluminum nanoparticles and submicroparticles: (**a**) Sample 1, (**b**) Sample 2, (**c**) Sample 3.

**Figure 5:**
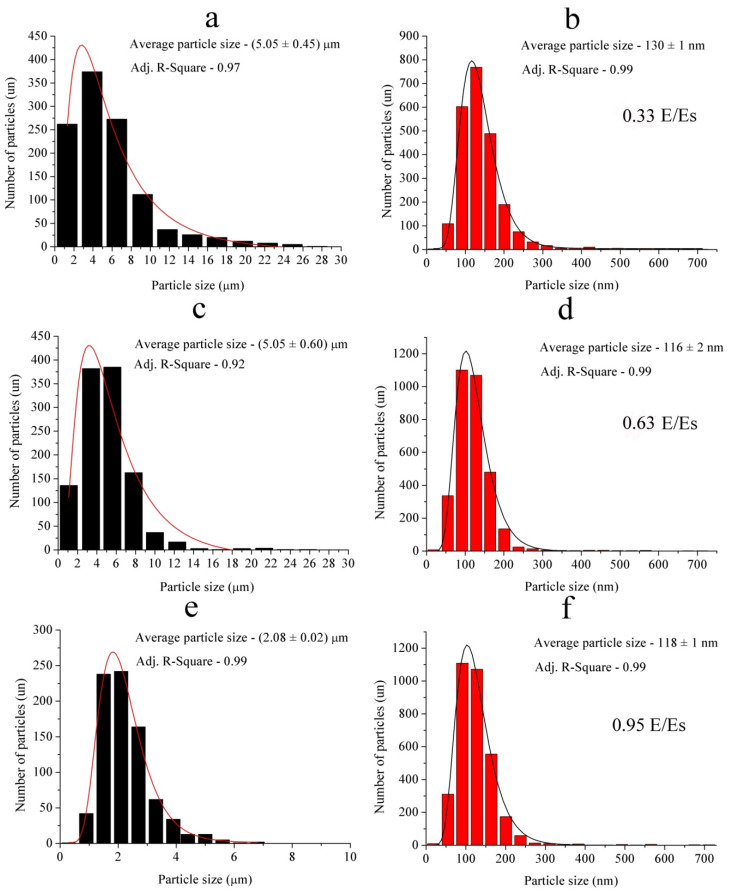
Microparticle (**a**,**c**,**e**) size distribution and nanoparticle and submicron particle (**b**,**d**,**f**) size distribution: (**a**,**b**) Sample 1, (**c**,**d**) Sample 2, (**e**,**f**) Sample 3.

The powders obtained consisted of the fine fraction including both nanoparticles and submicroparticles as well as coarse fraction including microparticles. Micrographs of all samples show that both nanoparticles and microparticles were spherically shaped (Figure 4). The micrographs show that the largest microparticles were present in Sample 1 (*E/E_s_* = 0.33). Sample 1 contained submicroparticles and microparticles larger than 10 μm in size (Figure 5a).

With an increase in energy input to *E/Es* = 0.63 (Sample 2), the proportion of submicron particles and the number of particles with a size from 5 μm to 8 μm increased, while the proportion of particles with a size of more than 10 μm decreased (Figure 4b and Figure 5c). An increase in energy input up to *E/Es* = 0.95 (Sample 3) led to a decrease in the average size of microparticles, while there were no particles larger than 10 microns (Figure 4c and Figure 5e).

In addition, the micrographs in Figure 4 show that along with nanoparticles (size less than 100 nm), the powders contained submicron particles with a size of more than 100 nm. Sample 1 (*E/Es* = 0.33) was represented by a relatively small proportion of nanoparticles; mainly by particles with a size more than 100 nm (Figure 5b). At *E/Es* = 0.63 (Sample 2), the proportion of nanoparticles increased, while the number of submicron particles decreased (Figure 5d). With an increase in energy to *E/Es* = 0.95 (Sample 3, Figure 5f), the above tendency persisted—that is, the proportion of nanoparticles increased, while that of submicron particles decreased. The average sizes of nano- and submicron particles for all samples were rather close, differing only in the ratio of the number of nano- and submicron particles. Additionally close were the average sizes of micron particles of Samples 1 and 2, while the average size of micron particles of the Sample 3 was markedly lower. Therefore, we believe that Sample 3 was markedly different from Samples 1 and 2.

Although the differences between nanoparticles and submicron particles obtained at different energies were less pronounced in comparison with microparticles, nevertheless, in this case, a decrease in the particle size was observed with an increase in *E/Es*. As follows from the data in Table 3, despite the fact that the aluminum overheating value when obtaining Samples 1 and 2 differed by 1.9 times, and by 1.5 times when obtaining Samples 2 and 3, the differences between Samples 1 and 2 were less pronounced than between Samples 2 and 3. This is probably due to the fact that the characteristics of the electrical explosion (*I/S* and *d*) of Sample 3 were close to the values of a uniform electrical explosion (Joule heating), as noted in the Reference [35]. The characteristics of the electrical explosion for Samples 1 and 2 corresponded to the explosion modes in which microscopic MHD instabilities develop.

**Figure 6:**
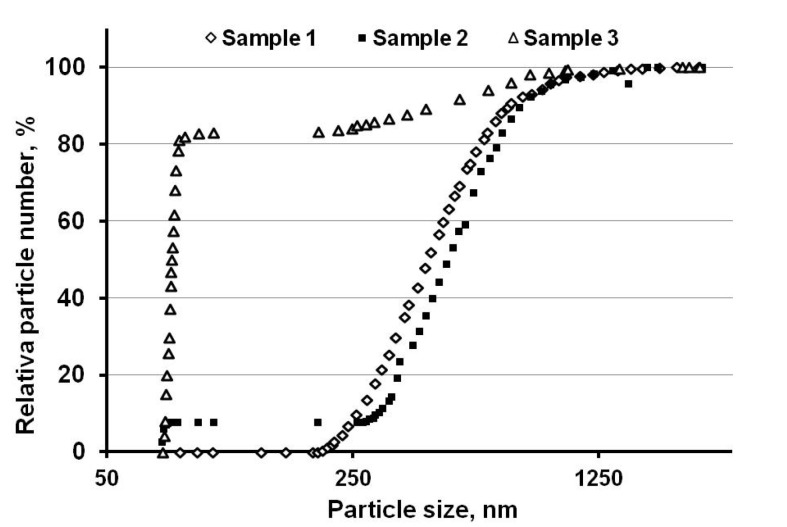
Integral particle size distribution of bimodal aluminum powders.

**Figure 7:**
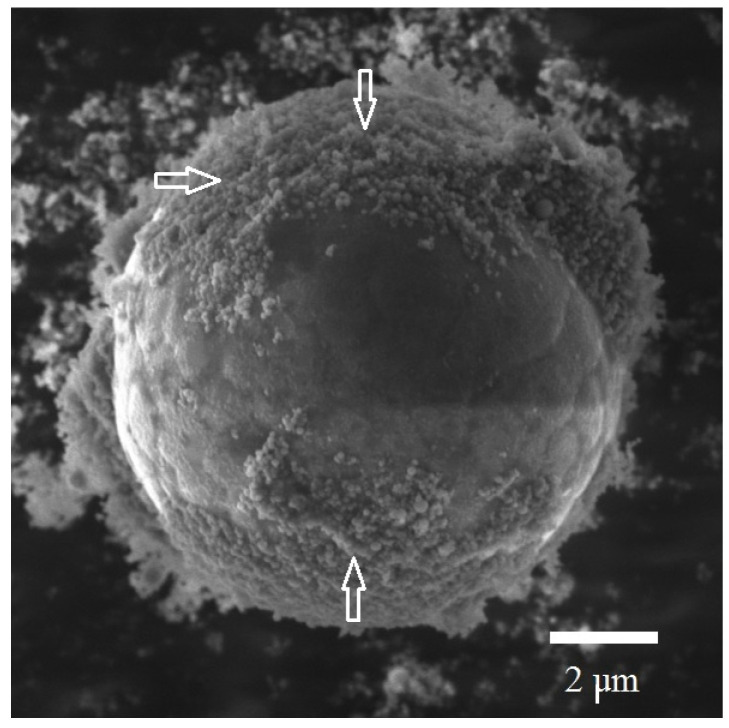
SEM image of nanoparticles and submicroparticles located on the surface of the microparticle (shown by arrows) (Sample 1).

Figure 6 shows that the relative number of nanoparticles in the samples was significantly different between them. Sample 1, obtained with a minimum value of *E/E_s_* = 0.33 did not show the presence of nanoparticles. At the same time, nanoparticles were present in the sample, as followed from electron microscopy analysis. In our opinion, this effect is due to the fact that there were a few nanoparticles in Sample 1. Therefore, nanoparticles along with submicroparticles were located on the surface of microparticles, as shown in Figure 7. The particle size distribution was determined by centrifugal sedimentation in a liquid medium; therefore, microparticles with nanoparticles with submicroparticles located on the surface were perceived as a single particle.

With an increase in the *E/E_s_* value, the number of nanoparticles in the sample increased; therefore, some of the nanoparticles were in a free state. In Sample 2, the proportion of such nanoparticles was about 8% of the total number of particles. In Sample 3 obtained at the maximum energy input, the number of such nanoparticles was about 82% of the total number of particles.

To make feedstock when manufacturing parts with complex shapes by such processes as MIM or MEAM (material extrusion additive manufacturing [41]), the powder is mixed with a polymer binder. An important characteristic of feedstock is flowability—otherwise, the viscosity of the feedstock in the molten state—which, other things being equal (given temperature, content of the dispersed solid component in the polymer matrix), is determined by the particle size as well as the particle size distribution of the dispersed solid phase [42]. Typically, feedstock contains particles with an average size between 5 and 15 µm [43] since feedstocks containing small particles tend to have a high viscosity [44]. On the other hand, the feedstock containing 12% stainless steel 316L nanoparticles, the rest being microparticles with a size of 4 μm, has been noted [45] to have a lower average viscosity compared with that containing only micropowder. This effect can be explained by the formation of the close-packed particle agglomerates strongly affecting the flow of the feedstock melt. Particle agglomerates occlude a certain amount of dispersion liquid in which the agglomerates are distributed. Accordingly, the effective volume of the particle agglomerates increases, and the volume of the dispersion liquid decreases, which entails a significant increase in the viscosity. Small particles in bimodal powders are distributed in the voids between large particles and displace part of the occluded dispersion liquid; the effective volume of the particle agglomerate does not change, while the volume of the dispersion liquid increases. This leads to a decrease in the viscosity of feedstock with bimodal powder compared with feedstock with monomodal powder. The maximum packing fraction of particles in the agglomerate depends on the ratio of the diameters of the particles making up the agglomerate [46]. Thus, the maximum degree of filling the agglomerate volume in case of monodisperse particles is 0.6435; for bimodal particles with a particle diameter ratio of 10, the maximum degree increases to 0.8270; with a further increase in the particle diameter ratio, the maximum filling degree can reach 0.871.

As follows from the Table 4, the maximum MFI value was observed for Feedstock 1 (microparticle to nanoparticle diameter ratio *R* value was 38.8, Table 3). Close MFI value was observed for Feedstock 2 (*R* = 43.5). Feedstock 3 showed a relatively low MFI value (*R* = 17.6). The minimum MFI value of Feedstock 4 containing only microparticles was lower than that of feedstock based on bimodal powders.

## 4. Conclusions

Electrical explosion of aluminum wire is a versatile method to obtain bimodal aluminum powders with a given nano- and microparticle size distribution. All other things being equal, the disperse composition of aluminum bimodal powders is determined by the level of energy input into the wire. As the energy input into the wire increases, the proportion of microparticles decreases, and the proportion of submmicroparticles and nanoparticles increases.

To obtain bimodal powders, it is preferable to destruct the wire, during which microscopic MHD instabilities develop. Nanoparticles in these electrical explosion modes will be formed from the vapor phase, and microparticles will be formed from liquid metal droplets.

To achieve high flowability of feedstock, it is necessary to provide the optimal ratio of particle diameters. For particle monomodal distribution in the powder used, the flowability of feedstock is relatively low. For the production of highly flowable feedstocks, the most promising are powders with at least a bimodal size distribution since fine particles fill voids among large particles, providing particle close packing and high filling degree of the feedstock with solid phase.

Bimodal aluminum powders obtained by the electrical explosion of wires can be a promising basis for the development of highly filled and highly flowable aluminum–polymer MIM and MEAM feedstocks.

## Figures and Tables

**Table 1:** Operating Parameters for Obtaining Al Powders.

Sample	*d*, mm	*l*, mm	*C,* µF	*L_in_,* µH	*U*_0_, kV
1	0.35	90	1.6	0.73	13
2	0.35	90	1.6	0.73	17
3	0.35	90	1.6	0.73	20

**Table 2:** Parameters of the Electrical Explosion of the Aluminum Wires *.

Sample	*I*, kA	*S*, cm^2^	*I/S*, A/cm^2^	*E/E_s_*
1	15.5	9.6 × 10^−4^	1.6 × 10^7^	0.33
2	19.5	9.6 × 10^−4^	2.0 × 10^7^	0.63
3	22.5	9.6 × 10^−4^	2.34 × 10^7^	0.95

* *I*—maximum value of the current *I(t)* flowing through the wire. *S*, wire cross sectional area; *I/S*, current density.

**Table 3:** Characteristics of the Particles Obtained at Different Energy Levels Input into Wires.

Sample	Average Size of Fine Fraction, nm	Average Microparticle Size, μm	Microparticle-to-Nanoparticle Diameter Ratio
1	130 ± 1	5.05 ± 0.45	38.8
2	116 ± 2	5.05 ± 0.60	43.5
3	118 ± 1	2.08 ± 0.02	17.6

**Table 4:** Melt Flow Index of feedstocks.

Feedstock	MFI, g/10 min
1	53.0
2	42.3
3	18.0
4 **	16.6

** Feedstock 4 was prepared with ASD-4 aluminum powder.

## Data Availability

Not applicable.
